# Clinical and Virological Efficacy of Etravirine Plus Two Active Nucleos(t)ide Analogs in an Heterogeneous HIV-Infected Population

**DOI:** 10.1371/journal.pone.0097262

**Published:** 2014-05-16

**Authors:** Luis F. López-Cortés, Pompeyo Viciana, José A. Girón-González, Alberto Romero-Palacios, Manuel Márquez-Solero, Maria A. Martinez-Perez, Miguel A. López-Ruz, Javier de la Torre-Lima, Francisco Téllez-Pérez, Marcial Delgado-Fernández, Milagros Garcia-Lázaro, Fernando Lozano, Mohamed O. Mohamed-Balghata, on behalf of Sociedad Andaluza de Enfermedades Infecciosas

**Affiliations:** 1 Instituto de Biomedicina de Sevilla (IBiS), Hospital Universitario Virgen del Rocío/CSIC/Universidad de Sevilla, Sevilla, Spain; 2 Hospital Universitario Puerta del Mar, Cádiz, Spain; 3 Hospital Universitario de Puerto Real, Cádiz, Spain; 4 Hospital Universitario Virgen de la Victoria, Málaga, Spain; 5 Hospital Universitario San Cecilio, Granada, Spain; 6 Hospital Universitario Virgen de las Nieves, Granada, Spain; 7 Hospital Costa del Sol, Málaga, Spain; 8 Hospital La Línea, Cádiz, Spain; 9 Hospital Universitario Carlos Haya, Málaga, Spain; 10 Hospital Universitario Reina Sofía, Córdoba, Spain; 11 Hospital Universitario de Valme, Sevilla, Spain; 12 Complejo Hospitalario de Jaén, Jaén, Spain; 13 Enfermedades Infecciosas, Microbiología y Medicina Preventiva, Hospital Universitario Virgen del Rocío, Sevilla, Spain; University of Rome Tor Vergata, Italy

## Abstract

Etravirine (ETV) is recommended in combination with a boosted protease inhibitor plus an optimized background regimen for salvage therapy, but there is limited experience with its use in combination with two nucleos(t)ide reverse-transcriptase inhibitors (NRTIs). This multicenter study aimed to assess the efficacy of this combination in two scenarios: group A) subjects without virologic failure on or no experience with non-nucleoside reverse-transcriptase inhibitors (NNRTIs) switched due to adverse events and group B) subjects switched after a virologic failure on an efavirenz- or nevirapine-based regimen. The primary endpoint was efficacy at 52 weeks analysed by intention-to-treat. Virologic failure was defined as the inability to suppress plasma HIV-RNA to <50 copies/mL after 24 weeks on treatment, or a confirmed viral load >200 copies/mL in patients who had previously achieved a viral suppression or had an undetectable viral load at inclusion. Two hundred eighty seven patients were included. Treatment efficacy rates in group A and B were 88.0% (CI_95,_ 83.9–92.1%) and 77.4% (CI_95_, 65.0–89.7%), respectively; the rates reached 97.2% (CI_95_, 95.1–99.3%) and 90.5% (CI_95_, 81.7–99.3), by on-treatment analysis. The once-a-day ETV treatment was as effective as the twice daily dosing regimen. Grade 1–2 adverse events were observed motivating a treatment switch in 4.2% of the subjects. In conclusion, ETV (once- or twice daily) plus two analogs is a suitable, well-tolerated combination both as a switching strategy and after failure with first generation NNRTIs, ensuring full drug activity.

**Trial registration:**

ClinicalTrials.gov NCT01437241

## Introduction

Etravirine (ETV), a second-generation non-nucleoside reverse transcriptase inhibitor (NNRTI), was designed to overcome common first-line NNRTI resistance mutations; it has demonstrated a potent activity in vitro and in vivo in short-term monotherapy trials both in naïve subjects and in patients with high levels of phenotypic resistance to efavirenz and nevirapine [1]–[3]. Based on the results of 2 randomized clinical trials (DUETs), ETV became approved (200 mg twice daily) for salvage therapy in combination with optimized background therapy including a ritonavir-boosted protease inhibitor (PI/rtv) [4]–[7].

ETV is not recommended to be administered with nucleos(t)ide reverse-transcriptase inhibitors (NRTIs). This recommendation is based mostly on the results of a phase II trial, which compared the efficacy of ETV against that of a protease inhibitor, where both treatments were administered in combination with two NRTIs after a first-line virologic failure in an NNRTI-based regimen. Nevertheless, the high rate of at least two baseline resistance mutations to the NRTIs and NNRTIs in the previous trial did not allow the researchers to obtain accurate conclusions [8].

In spite of its antiviral activity, its favorable pharmacokinetics for once-daily administration, its safety and drug interactions profile [9]–[15], there is very scarce information about the efficacy of ETV plus two NRTIs both in subjects without NNRTIs-resistance mutations and after a NNRTIs failure with limited resistance mutations [16]. In the first scenario, the Sense trial has evaluated, as a secondary objective, the efficacy of 400 mg ETV once daily vs. EFV plus two NRTIs in treatment-naïve patients up to 48 weeks; the primary objective was to assess neuropsychiatric tolerability at 12 weeks [17]. Two additional studies of switching in subjects with viral suppression but ongoing neuropsychiatric adverse events on EFV or toxicity under the previous regimen have also evaluated the efficacy of this combination for up to 24 weeks [18], [19]. In the second setting, only the long-term virologic responses in four patients with isolated K103N mutations have been reported [20].

In this study, we aim to evaluate the efficacy of an ETV plus 2 NRTIs regimen out of the context of advanced salvage therapy, where ETV is usually administered in combination with PI/rtv. Moreover, we have assessed if the efficacy of this regimen is independent of the once- or two-daily administration of ETV.

## Patients and Methods

### Study Population and Design

Adult HIV-infected subjects attending several HIV clinics from Andalusia (Spain) and switching to a regimen of ETV plus 2 NRTIs due to adverse events (AEs) or virologic failure (VF) on a preceding regimen were consecutively enrolled in this ambispective observational study from January 2009 (marketing authorization date for ETV in Spain) to September 2011 and followed up during 52 weeks. Patients were classified as group A (subjects without virologic failure on or no experience with NNRTIs switched due to AEs) and group B (subjects switched after a VF on an efavirenz- or nevirapine-based regimen). The NRTIs prescribed as part of HAART were selected by the responsible physicians on the basis of previous antiretroviral treatments (ART) and/or genotypic resistance testing. In the cases of a previous VF, the genotypic resistance tests had to demonstrate susceptibility or low-level resistance both to NRTIs (score <30; HIV Drug Resistance Database of Stanford University [21] and to ETV (score ≤2 in the weighted genotypic scoring algorithm from Vingerhoets et al. [22]); this weighted genotypic score assigns the following mutations’ score for ETV: Y181I/V: 3; L100Y/P, Y181C, or M230L: 2,5; V106I, E138A, V179F, or G190S: 1.5; V90I, A98G, K101E/H, V179D/T, or G190A: 1. The K103N mutation was not considered to influence the ETV susceptibility. HLAB57*01 testing was required for abacavir use. No other exclusion criteria were established except for pregnancy and concomitant use of drugs or non-prescription traditional or herbal medications, which might have had interactions with ETV pharmacokinetics [7].

### Ethics Statement

The patients provided verbal informed consent only, recorded in the clinical history, according to the Spanish regulation as the prescription of ETV plus 2 NRTIs was previous to the inclusion of the patients in the study and not conditioned to it. The study was conducted according to the Declaration of Helsinki guidelines, approved by a central ethics committee (Comité Autonómico de Ensayos Clínicos, Consejería de Salud, Junta de Andalucía) and the Spanish Agency for Medicine and Healthcare Products which approved this consent procedure, and registered at ClinicalTrials.gov (NCT01437241).

### Follow-up, Assessments and Endpoints

Patient assessments were performed at baseline and every three months thereafter, including AEs, biochemical and hematologic profiles; the CD4^+^ T cell count was measured by flow cytometry, and the plasma HIV-RNA was measured using the Roche Amplicor HIV-1 Monitor assay (version 1.5, Roche Diagnostic Systems). The primary endpoint was efficacy at 52 weeks assessed by -intention-to-treat analysis; regimen failure was defined as treatment interruption for any reason or VF. VF was defined as the inability to suppress plasma HIV-RNA to <50 copies/mL after 24 weeks of treatment, or a confirmed viral load >200 copies/mL in patients who had previously achieved a viral suppression or had an undetectable viral load at inclusion. The secondary outcomes included efficacy according to on-treatment analysis, changes in CD4^+^ cell counts, the incidence of AEs and lipid profiles. Hepatotoxicity was classified according to the highest aminotransferases level observed during therapy with respect to the upper limit of normal (ULN) (grade 0 (<1.25 ULN), grade 1 (1.25–2.5×ULN), grade 2 (2.6–5×ULN), grade 3 (5.1–10×ULN), and grade 4 (>10×ULN) or relative to baseline values in patients with chronic viral hepatitis or cirrhosis (grade 0 (<1.25×baseline), grade 1 (1.25–2.5×baseline), grade 2 (2.6–3.5×baseline), grade 3 (3.6–5×baseline), and grade 4 (>5×baseline)). Patients missing two consecutive scheduled visits were considered to be lost to follow-up.

### Statistical Analysis

Descriptive statistics were used for demographic, epidemiological and clinical data, prior ARTs, CD4 cell count and viral load. Kaplan–Meier plots were produced for the ‘time to event’ analyses, and comparisons between the treatment groups were made using the log-rank test. Chi-square tests and Spearman’s rank-correlation coefficients were used to assess the relationship between VF and qualitative and quantitative variables, respectively. Statistical calculations were performed with Statistical Product and Service Solutions software (version 19.0, SPSS, Chicago, IL, USA).

## Results

### Baseline Patients’ Characteristics

A total of 346 patients started a regimen of ETV plus two NRTIs in the study period. Fifty nine of them were excluded from the analysis to homogenize the analysis population (naïve patients, 20; restarting ART after losing follow-up, 29; NRTI mutations conferring resistance to prescribed analogs, 10). The baseline characteristics of the 287 analyzed subjects are shown in table 1. Notably, more than one third of the subjects had chronic hepatitis (n = 77; 26.8%) or cirrhosis (n = 26; 9.0%).

**Table 1:** Baseline characteristics of the patients.

	Group A	Group B
	(n = 242)	(n = 45)
Male, no. (%)	183 (75.6)	34 (75.6)
Age, years, M (range)	44 (21–81)	42 (25–67)
Weight, kg, M (range)	69 (45–139)	67 (49–116)
Risk factor for HIV, no. (%)		
Previous iv drug use	80 (33.0)	14 (31.1)
Hetero/homosexual	154 (63.6)	29 (64.4)
Other	8 (3.3)	2 (4.4)
Methadone treatment, no. (%)	30 (12.3)	8 (17.7)
Nadir CD4/µl, M (range)	198 (4–762)	218 (6–480)
Clinical category C, no. (%)	57 (23.5)	9 (20.0)
Chronic hepatitis/cirrhosis, no. (%)	69 (28.5)/21 (8.6)	8 (17.7)/5 (11.1)
HCV	81 (90.0)	9 (69.2)
HBV	5 (5.5)	1 (7.6)
HCV+HVB	−	2 (14.3)
Other	3 (3.3)	2 (14.3)
No. previous ART, M (range)	2 (1–9)	2 (1–7)
Months on previous ART, M (range)	36 (1–186)	37 (3–168)
Previous antiretroviral regimen, no. (%)		
EFV +2 NRTIs	134 (55.3)	38 (84.4)
NVP +2 NRTIs	16 (6.6)	7 (15.5)
IP/rtv +2 NRTIs	87 (35.9)	−
3 NRTIs	5 (2.0)	−
Previous failure on NNRTIs, no. (%)	−	45 (100)
Available genotypic resistance test, no. (%)	137 (56.6)	45 (100)
Wild type	124 (90.5)	17 (37.8)
K103N	−	22 (48.9)
L100I or K101E or V179T	−	3 (6.6)
G190A	−	2 (4.4)
CD4/µl, M (range)	473 (12–1360)	468 (31–1351)
HIV-RNA copies/ml, M (range)	<50 (<50–444867)	982 (108–170000)
HIV-RNA <50 copies/ml, no. (%)	179 (74.0)	−
Associated NRTIs, no. (%)		
TDF+FTC	146 (60.3)	22 (48.9)
ABV +3TC	83 (34.3)	9 (20.0)
AZT+TDF	5 (2.1)	10 (22.2)
ABV+TDF	5 (2.1)	2 (4.4)
AZT +3TC	2 (0.8)	−
ddI +3TC	1 (0.4)	2 (4.4)
ETR 400 mg qd, no. (%)	215 (88.8)	29 (64.4)

ART: antiretroviral treatment. NRTIs: nucleos(t)ide reverse-transcriptase inhibitors. NNRTIs: non-nucleoside reverse-transcriptase inhibitors. EFV: efavirenz. NVP: nevirapine. TDF: tenofovir. FTC: emtricitabine. ABV: abacavir. 3TC: lamivudine. AZT: zidovudine.

### Switching to an ETV-based Regimen Due to Toxicity (Group A)

**Figure 1:**
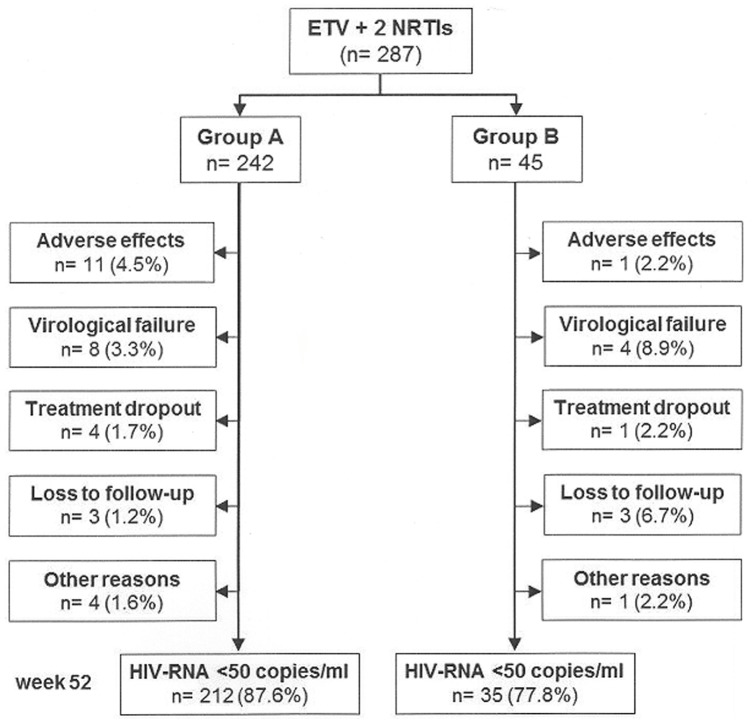
Patient disposition. ETV: etravirine. NRTIs, nucleos(t)ide reverse-transcriptase inhibitors. Group A: subjects without virologic failure on or no experience with non-nucleoside reverse-transcriptase inhibitors switched due to adverse effects. Group B: subjects switched after a virological failure on an efavirenz- or nevirapine-based regimen.

In this group, 242 subjects (84.3%) without experience with or VF on NNRTIs were switched to an ETV-based regimen due to AEs during their previous regimen (NNRTIs-based, 150; PI/rtv-based, 84; other, 8). ETV was dosed at 400 mg once daily in 88.8% of the patients. Most patients received tenofovir plus emtricitabine (60.3%) or abacavir plus lamivudine (34.3%). Seventy four per cent (n = 179) showed an undetectable HIV viral load at the time of switching to an ETV-based regimen. The remaining 63 subjects had a median viral load of 720 copies/mL (range, 69–444867) as treatment switches occurred before achieving an undetectable viral load with the previous regimens. The Kaplan–Meyer estimates for efficacy at 52 weeks were 88.0% (CI_95_, 83.9–92.1%) for the intention-to-treat analysis and 97.2% (CI_95_, 95.1–99.3%) for the on-treatment analysis. Thirty subjects (12.4%) had treatment failures, out of whom only 8 (3.3%) were due to VF; the remaining failures were due to AEs (n = 11; 4.5%), treatment dropout (n = 4; 1.7%), loss to follow-up (n = 3; 1.2%), death (n = 2; 0.8%. Non-Hodgkin lymphoma and metastatic melanoma, respectively), AEs attributable to concomitant peginterferon plus ribavirin therapy (n = 1; 0.04%) and imprisonment (n = 1; 0.04%) (figure 1). The median increase in CD4^+^ cell counts from baseline to week 52 was 58 cells/µL (IQR: –15 to 198) and was inversely proportional to baseline CD4^+^ counts (r = –0.244; p<0.001).

### Switching to an ETV-based Regimen Due to Virologic Failure on a First-generation NNRTI (group B)

Forty five patients (15.6%) were included in this group after failing an efavirenz- or nevirapine-based regimen. Before switching, genotypic resistance tests showed the following results: i) a wild-type strain in 17 subjects, ii) an isolated K103N mutation in 14, iii) the K103N plus one other NNRTI mutation in 7 (L100I; K101E; V179I/T; Y188L; G190A, and P225H, respectively), and iv) other NNRTI mutations (without the K103N) in another 7 subjects (L100I; G190A; P225H; K101Q+V108I; K101R+K103T; V106I+V179D; V108D, P225H). Thus, no subjects showed an ETV score >2 at baseline. Additionally, 5 patients had NRTI mutations conferring low-level resistance to one of the NRTIs included in the regimen. ETV was dosed at 400 mg once daily in 64.4% of the subjects.

In group B, the Kaplan–Meyer estimates for efficacy at 52 weeks were 77.4% (CI_95_, 65.0–89.7%) for the intention-to-treat and 90.5% (CI_95_, 81.7–99.3) for the on-treatment analysis, being slightly lower than the efficacy found in group A (p = 0.006 and 0.054, respectively). The reasons for treatment failure were as follows: VF (4; 8.9%), treatment drop-out or loss to follow-up (n = 4; 8.9%), AEs (n = 1; 2.2%) and ETV interaction with rifampin (n = 1; 2.2%) (figure 1). The median CD4^+^ cell count increase from baseline to week 52 was 61 cells/µL (IQR: –6 to 156), inversely proportional to baseline CD4^+^ counts (r = –0.367; p = 0.039).

### Variables Associated with Virologic Failures

The VF episodes were more frequent in group B (4/45) than in group A (8/242), although the difference was not statistically significant (8.9% vs. 3.3%; p = 0.101). No relationships were found between a poor virologic outcome and the baseline ETV score, the presence of mutations conferring low-level resistance to the NRTIs or the NRTIs combination used. Among the patients from group A, the VF rates for a once- and twice-daily administration were 2.8 vs. 6.9%, respectively (p = 0.243); for the subjects in group B, these values were 3.4 vs. 21.4%, respectively (p = 0.094).

Plasma HIV-RNA amplification was achieved in 11 out of these 12 patients with VF. Four of these 11 patients exhibited a wild-type virus while the remaining 7 subjects showed 1 or more new NNRTI mutations. Additionally, 4 out of these 7 subjects with new NNRTI mutations also showed new NRTI mutations (table 2).

**Table 2:** Genotypic resistance tests at virological failure.

		Previous mutations in RT				Mutations in RT at virological failure			
	Failure on	Analogues	Non-analogues	ETV	Treatment	Analogues	Non-analogues	ETV	VL at VF
	NNRTIS			score*				score*	
1	yes	K219Q	K103N, Y188L	0	AZT,TDF,ETV	K219Q, 69D, K70R	K103N,V179I,Y188L,H221Y	0	3660
2	yes	−	K103N, V179T	0	AZT,TDF,ETV	−	A98G,L100I,K103N,V179T	4.5	11900
3	no	M184V	−	0	AZT,TDF,ETV	M41V	Y181C	2.5	2570
4	no	wt		0	TDF,FTC,ETV	A62V, K65R, M184I	K101E,V179F,Y181C,G190A	6	612
5	no	wt		0	TDF,FTC,ETV	K65N, Y115F, M184V	A98G,L100I,E138K	3,5	308
6	no	wt		0	ABV,3TC,ETV	−	E138G,V179F,Y181C	4	85700
7	no	wt		0	ABV,3TC,ETV	−	L100I	2,5	1650
8–9	no	wt		0	TDF,FTC,ETV	wt		0	692–2650
10–11	no	wt		0	ABV,3TC,ETV	wt		0	103000–817751

RT, reverse transcriptase. wt: wild-type. VL, viral load. VF, virogical failure.

*Mutation scoring according to reference 22.

### Safety

**Figure 2:**
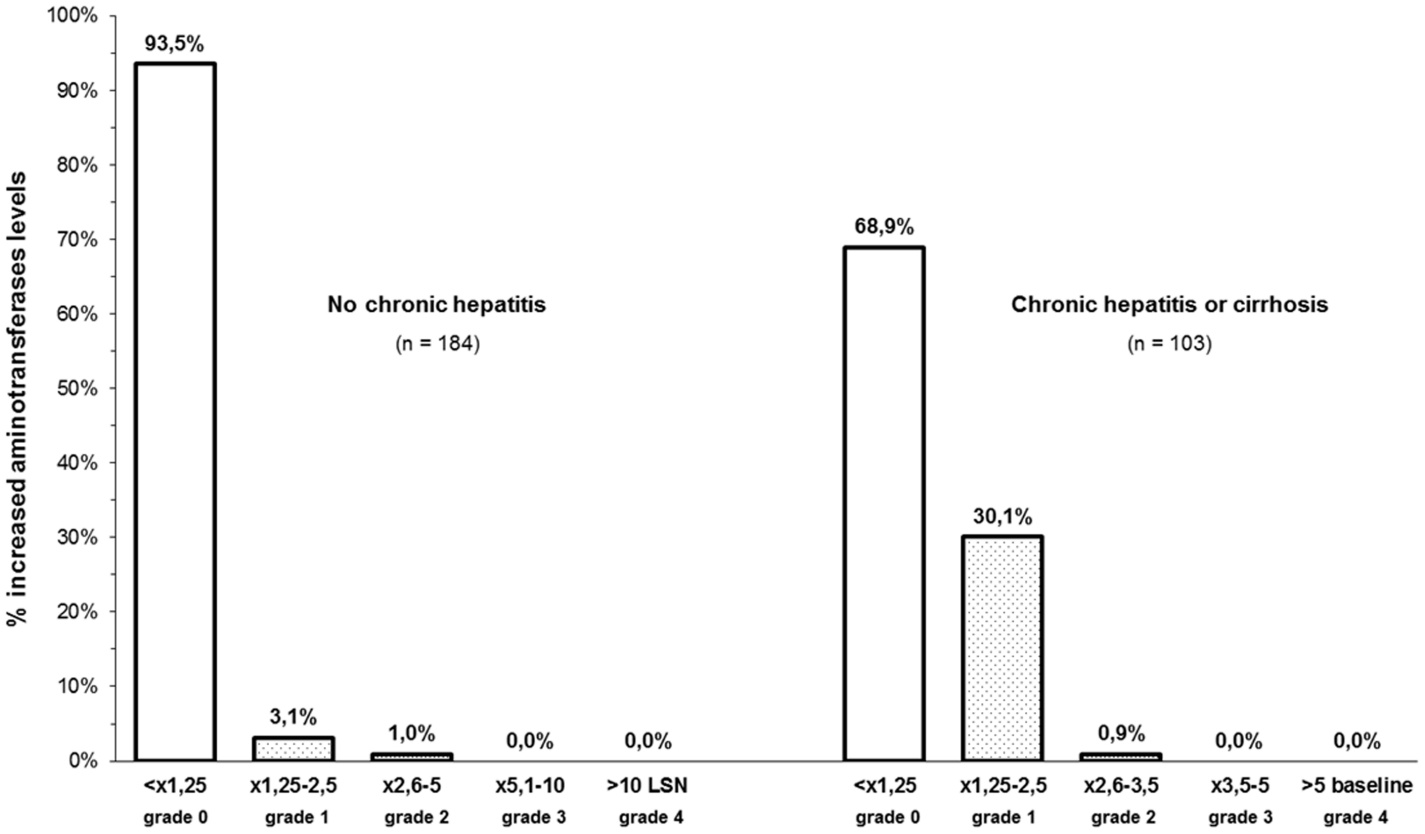
Proportion of patients with increased aminotransferases levels at any time-point throughout the follow-up.

Forty one patients (14.3%) reported AEs (rash, 6; nausea or vomiting, 9; diarrhea, 4; constipation, 2; abdominal discomfort, 1; dizziness, 9; nightmares or insomnia, 5; depression, 2; peripheral neuropathy, 1; lipoatrophy, 1; lipohypertrophy, 1). Although these AEs were grade 1–2 in all cases, they motivated a treatment switch in 12 subjects (4.2%) (nausea or vomiting 4, and 1 case of each of the following: rash, constipation, depression, dizziness, hypercholesterolemia, peripheral neuropathy, general discomfort, and lipoatrophy). However, figure 2 shows the limited proportion of patients with increased aminotransferase levels at any time-point throughout the follow-up; none of these cases were symptomatic, and the alterations observed were transient and improved without treatment discontinuation in all cases.

**Figure 3:**
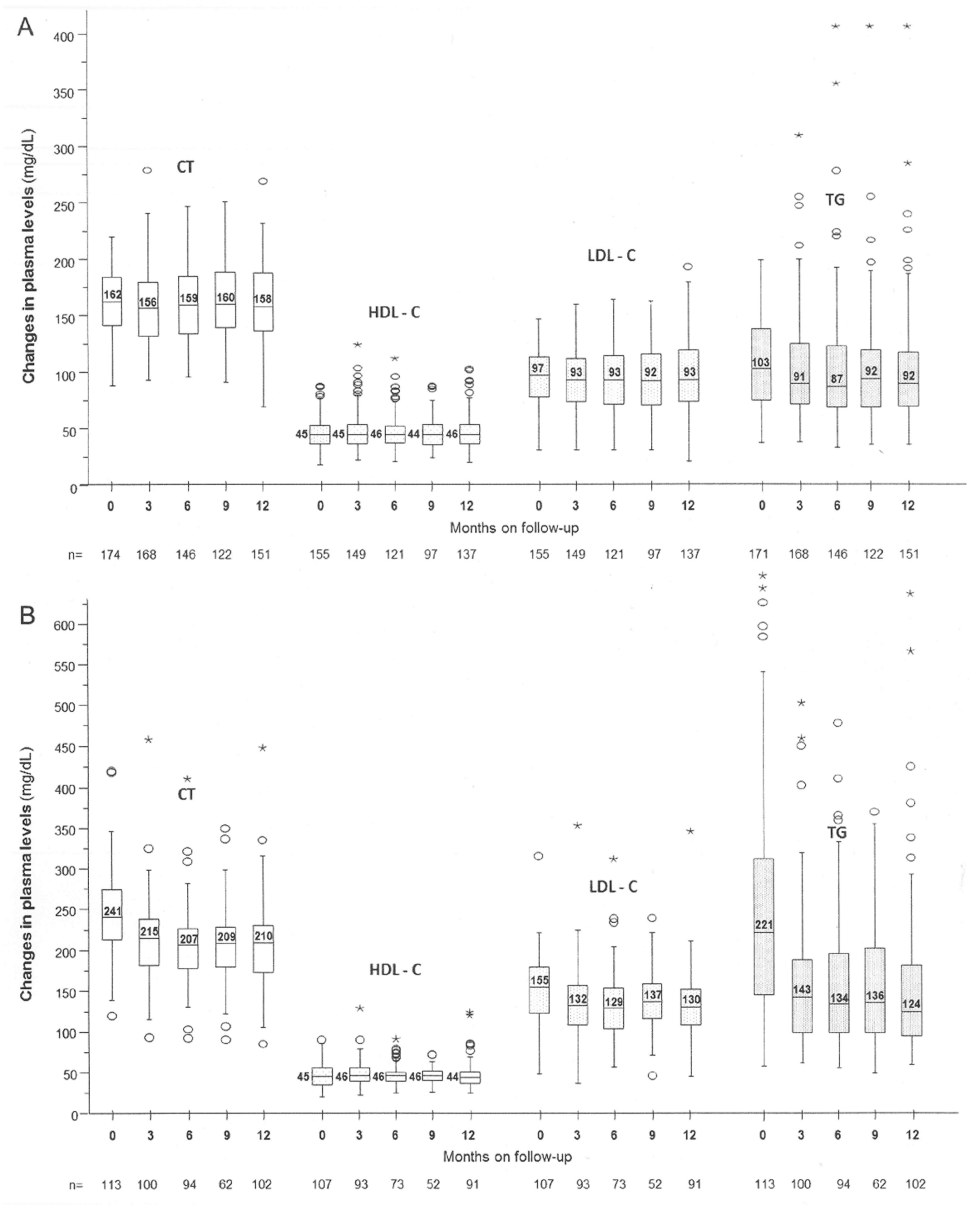
Change in lipid plasma levels (mg/dL) throughout the follow-up. A: patients with normal baseline values. B: patients with abnormal baseline values on previous NNRTIs- or PI/rtv-based regimens. CT: total cholesterol. HDL-C: high-density lipoprotein cholesterol. LDL-C: low-density lipoprotein cholesterol. TG: triglycerides.

As many of the patients switched to ETV due to lipid alterations induced by the preceding antiretroviral regimens, the evolution of lipid profiles was analyzed according to whether the patients had normal (total cholesterol and triglycerides of ≤220 and 200 mg/dL, respectively) or abnormal baseline lipid levels to avoid skewing the results. Thereby, the 151 subjects with normal baseline lipid profiles completed 52 weeks on therapy with median changes in total cholesterol (TC), low-density lipoprotein cholesterol (LDL-C), high-density lipoprotein cholesterol (HDL-C) and triglycerides (TG) levels of –3 mg/dL (IQR, –21 to 15. P_90_, 36; p = 0.341), 0 mg/dL (IQR, –5 to 13. P_90_, 13; p = 0.771), –2 mg/dL (IQR, –14 to 11. P_90_, 28; p = 0.612) and –3 mg/dL (IQR, –30 to 20. P_90_, 43; p = 0.054), respectively (figure 3A). Lipid abnormalities above the upper limit of normality were observed in 11 subjects; in 10 out of these 11 subjects the abnormalities were grade 1 (TC, 222–269 mg/dL in 7 subjects; TG, 226–406 mg/dL in 4 subjects).

Moreover, 107 patients switched to an ETV-based regimen due to lipid alterations while on the earlier antiretroviral regimen (60% on EFV; 38% on PI/rtv). A lipid profile was available in 91 of the subjects after 52 weeks on therapy, with the corresponding changes in TC, LDL-C, HDL-C and TG levels of –32 mg/dL (IQR, –61 to –8. P_90_, 16; p<0.001), –1 mg/dL (IQR, –8 to 7. P_90_, 13; p = 0.505), –19 mg/dL (IQR, –42 to –2. P_90_, 28; p<0.001) and –68 mg/dL (IQR, –154 to –17. P_90_, 21; p<0.001), respectively (Figure 3B). Additionally, only 1 out of the 48 patients on methadone treatment reported symptoms of methadone withdrawal.

## Discussion

Our study focused on the clinical efficacy of ETV plus two active NRTIs in two well-defined scenarios in which there is little experience with the use of this antiretroviral combination: 1) subjects without resistance to NNRTIs who experienced AEs during an NNRTIs- or a PI/rtv-based regimen, and 2) as a rescue regimen after a VF on a first-generation NNRTI-based regimen.

In the first setting, the efficacy at 52 weeks was striking; the AEs that motivated switching from the preceding regimen were resolved in most cases, and the virological success rate (97.2%) was comparable to the best results of the clinical trials on ART. This figure is also remarkable when taking into account that these results were based on routine clinical practice. It seems reasonable to assume that the high genetic barrier of ETV to the development of resistance may have contributed to these favorable results. In the second scenario, we observed a VF rate of only 8.9%.

ETV was administered as a once-daily dosing regimen in most subjects. Three pharmacokinetic studies have shown a lower ETV C_min_ in both healthy volunteers and HIV-infected patients who were administered ETV 400 mg once daily, as compared to 200 mg twice daily; nevertheless, the ETV C_min_ in the once daily patients was still well above the protein binding adjusted EC_50_ for wild type HIV. Moreover, similar intracellular ETV levels were observed in HIV-infected patients with both dosing regimens [9]–[11]. No relationship was identified between the dosing regimen and the efficacy in the group A but an association trend was identified between the dosing regimen and the efficacy in the group B in which the VF rate was higher with the twice daily than with the once-daily administration (3.4 vs. 21.4%; p = 0.094). Therefore, we believe that a worse adherence with the twice daily regimen may have influenced these results. Although this finding supports the routine once daily administration of ETV in the absence of NNRTIs-resistance mutations that significantly impact ETV susceptibility, a larger study is needed to confirm our results in the second scenario.

Regarding the genotypic resistance tests of the 11 patients with VF, resistance-associated mutations which significantly decrease ETV susceptibility appeared in 5 out of the 11 patients (45%); wild-type strain or minor resistance mutations, which do not significantly decrease ETV susceptibility, were observed in the remaining subjects. New NRTI mutations only appeared in four out of the 11 subjects. The high viral load levels observed in some VF may suggests a low adherence.

Regarding the safety profile of this combination, no clinical grade 3 or 4 AEs were recorded. In fact, most of the treatment changes due to AEs, some of which were questionably related to either of the study drugs, would reflect the current widespread availability of antiretroviral drugs rather than the patient’s AE severity. The incidence of mild-to-moderate rash (2.0%) was lower than previously reported [6], and the transient grade 1 transaminase increases among the patients with chronic viral hepatitis and/or cirrhosis could be due to the natural evolution of chronic hepatitis rather than to pharmacological toxicity. Likewise, the incidence of abnormalities in lipid parameters among those patients with normal baseline values was negligible, and there was a substantial improvement in lipid profiles after switching to an ETV-based regimen in those patients with abnormal baseline values, as has been previously observed [6], [19].

We believe that our study has two main limitations. Firstly, there were a limited number of subjects included in group B; however, given that minimal data are available for this scenario, our results may allow clinicians to consider this therapeutic option after a VF on a first-generation NNRTI-based regimen with limited resistance mutations. Secondly, adherence was not objectively measured. A low compliance might have negatively influenced the treatment efficacy rate, particularly in those subjects with a previous VF for whom poor medication adherence might have been the cause of both the previous and the actual failures. However, we believe that our work accurately reflects the efficacy of treatment with ETV plus 2 NRTIs in routine clinical practice in two scenarios where the treatment options are not well-defined.

In summary, ETV plus two analogs is a suitable, well-tolerated combination both as a switching strategy due to the toxicity of NNRTIs or PI/rtv-based regimens and after failing with first-generation NNRTIs, ensuring full drug activity. In these scenarios, a once daily administration of ETV is as effective as a twice daily regimen and would enhance regimen compliance.

In summary, ETV plus two analogs is a suitable, well-tolerated combination both as a switching strategy due to the toxicity of NNRTIs or PI/rtv-based regimens and after failing with first-generation NNRTIs, ensuring full drug activity. In these scenarios, a once daily administration of ETV is as effective as a twice daily regimen and would enhance regimen compliance.
